# FOXM1 is regulated by DEPDC1 to facilitate development and metastasis of oral squamous cell carcinoma

**DOI:** 10.3389/fonc.2022.815998

**Published:** 2022-08-22

**Authors:** Jing Qiu, Yongping Tang, Lan Liu, Jiangbo Yu, Zhenggang Chen, Hao Chen, Rongtao Yuan

**Affiliations:** ^1^ Department of Stomatology, Qingdao Municipal Hospital, Qingdao, Shandong, China; ^2^ Key Laboratory of Biomedical Information Engineering of Ministry of Education, Biomedical Informatics & Genomics Center, School of Life Science and Technology, Xi’an Jiaotong University, Xi’an, Shanxi, China; ^3^ Research Institute of Xi’an Jiaotong University, Hangzhou, Zhejiang, China

**Keywords:** oral squamous cell carcinoma, proliferation, migration, DEP domain containing 1, FoxM1

## Abstract

The Disheveled, EGL-10, Pleckstrin domain containing 1 (DEPDC1) is a new oncogene that has recently been described. The mechanisms and functions of its expression are yet to be determined in oral squamous cell carcinoma (OSCC). In the present study, the impact of DEPDC1 on the growth and development of OSCC was investigated using animal models, cell lines and human tissue samples. Elevated DEPDC1 expression within cancer cell lines and human OSCC has been identified. Mechanistic examination showed that restored DEPDC1 expression *in vivo* and *in vitro* stimulated OSCC tumour development. In addition, FOXM1 interacts with DEPDC1 as indicated by co-immunoprecipitation and immunofluorescence testing. Functionally, DEPDC1 facilitated Wnt/β-catenin signal transduction and β-catenin protein nuclear expression. In summary, the DEPDC1, interacting with FOXM1 *via* Wnt/β-catenin signaling, the closely regulated OSCC pathogenesis, suggesting that targeting the novel DEPDC1/FOXM1/β-catenin complex is an essential OSCC therapeutic approach.

## Introduction

An increasing malignancy worldwide, head and neck squamous cell carcinoma (HNSC) comprises 90% of all head and neck cancers (1). The primary subtype of HNSCs is oral squamous cell carcinoma (OSCC), one of the most common cancers worldwide (1, 2). Statistics for oral cancer are unreliable compared to other cancers because half of all oral cancers are not detected until cancer has spread to nearby tissues and around half of the patients die within 5 years (3, 4). With over 90% of patients diagnosed with squamous cell carcinomas (SCC), most oral cancer originates from oral cavity neoplasms. OSCC therapeutic strategies include chemotherapy, radiation therapy and surgery (5). Despite the disease’s alarming death and morbidity statistics, OSCC is one of the least studied malignancies, with limited understanding of its molecular pathophysiology. Hence, detailed studies must be carried out to identify the inherent mechanisms underlying tumour incidence to unveil potential OSCC therapies. DEPDC1 is a newly presented and well-preserved oncogene from mammals to *Caenorhabditis elegans* (6, 7). N terminal regions contain the DEP domain, and the gene for DEPDC1 is found at 1p31.3 in humans (6). DEPDC1 was first discovered at an abnormally raised expression in bladder cancer. It inhibited A20 transcription by associating with zinc-finger protein 224 (ZNF224), activating the anti-apoptotic pathway and stimulating the NF-κB pathway (8).

DEPDC1 is abnormally over-expressed in the prostate, hepatic, breast, and lung cancers, according to recent research, and can predict outcomes in lung and bladder cancer patients (9–12). Its activities in promoting tumour growth have also been gradually uncovered. DEPDC1 is primarily expressed throughout the cell cycle’s interphase stage. It is necessary for normal metaphase division, as shown by drastic mitotic arrest when silencing it (7). Moreover, DEPDC1 regulates chemotherapy agents targeting microtubules to induce apoptosis by improving BCL-2 family protein MCL1’s JNK-dependent degradation (6). Another study revealed that knocking down DEPDC1 caused a delay in nasopharyngeal cancer cell cycle progression, proliferation, and substantial migratory inhibition (13). However, in OSCC progression and growth, the functions and processes underlying DEPDC1 expression are yet to be elucidated. Forkhead box M1 (FOXM1) is a FOX superfamily transcription factor with a preserved DNA binding domain in the form of a winged helix (14).

Several studies have demonstrated that it plays a critical role in exacerbating metastatic activity, invasion, angiogenesis and cancer since FOXM1 is a crucial cell cycle proliferation regulator (15–17). We recently discovered that FOXM1’s down-regulation prevents OSCC’s invasion, migration and replication *in vitro* (17). In the nucleus, FOXM1 can trigger the expression of several genes that are involved in tumor initiation processes such as angiogenesis, cell proliferation, cellular migration and invasion (18). FOXM1 also synergizes with the canonical Wnt signaling pathway (often activated during tumorigenesis) by directing the nuclear translocation of β-catenin to induce transcription of several oncogenes (18). Additionally, increased FOXM1 expression induces changes in the methylation status similar to the epigenome in OSCC. Among those involved in OSCC progression and development, Wnt/β-catenin is one of the most efficient signaling mechanisms (19). Wnt ligands interact with Frizzled family receptors and cell surface co-receptors, blocking this pathway. Ligand binding inhibits the cytoplasmic degradation complex, including casein kinase 1, Axin, glycogen synthase kinase-3β, and adenomatous polyposis coli (APC) leading to β-catenin nuclear localization. Vector T-cell factor/lymphoid-enhancing factor is bound by β-catenin inside the nucleus to trigger downstream Wnt target genes, including matrix metalloproteinase 9 (MMP9), MMP2 and cyclin D1. Despite evidence indicating that Wnt/β-catenin signals alone are insufficient to cause cancer, such signaling has played a crucial role in the survival, chemoresistance, and development of cancer stem cells (20). FOXM1 is a relevant target for further characterization because FOXM1 regulates the expression of many genes and affects epigenetic controls that are involved in multiple oncogenic cellular processes. FOXM1 promotes the Wnt/β-catenin signaling pathway by promoting β-catenin nuclear translocation, according to newly published research (21).

Nevertheless, the biomechanical processes mediating that suppression and its role in the relationship between β-catenin and DEPDC1 remains unclear. The mechanisms and roles of DEPDC1 expression in OSCC migration and growth were determined in this study. We also observed that DEPDC1 expression was increased in OSCC cells and that DEPDC1 downregulation impaired the growth of OSCC cells and *in vivo* and *in vitro* metastasis. DEPDC1 downregulation is the cause of DEPDC1 interacting with FOXM1 and lowering the nuclear translocation of β-catenin.

## Materials and methods

### Collection of tumorous tissue

At the Qingdao Municipal Hospital, 42 related samples of tumorous tissue (central area of the lesion) and matched adjoining oral epithelial tissue were obtained post-surgically from patients with OSCC. All patients provided informed consent before sampling and had not received any treatment before surgery. All of the samples were kept at -80°C. The Ethical Committee of Qingdao Municipal Hospital authorized and approved this research (approval number 2018-0318).

### Cell culture and cell transfection

The American Type Culture Collection (ATCC) supplied SCC-15, SCC-25, HaCaT, HEK29T and CAL-27 cell lines which were cultured in RPMI-1640 with streptomycin (100 μg/mL) (Solarbio, China), penicillin (100 U/mL) and 10% of fetal bovine serum (Gibco, USA). Overexpression vector-DEPDC1 (OV-DEPDC1), sh-DEPDC1, sh-FOXM1, OV-DEPDC1+ sh-FOXM1, OV-DEPDC1+ sh-NC, OV-NC + sh-FOXM1, OV-NC+ sh-NC and negative control of the overexpression vector (OV-NC) were transfected into the cells. The GeneChem Company (Shanghai, China) developed these plasmids. The cells were grown on a 6-well plate for 48h and transfected upon reaching 70-80% confluent. The transfection was performed using an assay based on lipofectamine 3000 (Thermo Fisher Scientific, Massachusetts, USA). The medium was removed following an 8-hour transfection, and the cells were incubated for the specified times, then extracted and used for the designated experiments.

### Bioinformatics analyses

Comparative investigations of DEPDC1 expression profiles were performed using multiple web-based bioinformatics techniques. The datasets from Gene Expression Omnibus (GEO, access #: GSE31056) were downloaded, assessed and used for calculations to obtain stored expression data and systematically interpret and combine datasets. GraphPad Prism software was utilized for mapping the expression profile. Gene Expression Profiling Digital Research (GEPIA, http://gepia2.cancer-pku.cn/) is a well-known tool for examining variations between cancerous and paired normal tissue mRNA levels expressed from particular genes (22). GEPIA was used to study mRNA levels in HNSC and paired normal tissues. UALCAN (http://ualcan.path.uab.edu/) was employed to test the DEPDC1 gene prognosis (23). Relative to normal RNA expression, cancer patients were classified into low and high expression groups for each gene, and the probability threshold P < 0.05 was considered significant for each difference. The OncoLnc (http://www.oncolnc.org/) database was first utilized to evaluate the prognostic value of DEPDC1 mRNA expression in HNSC (24).

### RNA isolation and quantitative real-time polymerase chain reaction

Using TRIzol reagent (Thermo Fisher Scientific, Massachusetts, USA), we isolated whole-cell RNA from cell lines, normal tissues, and tumours (17). Takara (Shiga, Japan) supplied the PrimeScript RT Reagent Kit to generate cDNA at a 20 μL volume following manufacturer instructions. The cDNAs were subsequently used in qRT-PCR using SYBR^®^ Premix Ex Taq™ kits according to manufacturer’s instructions (Takara, Japan). QRT-PCR was performed in a FTC-3000 (Funglyn Biotech, Canada). The forward PCR primer used for DEPDC1 (225 bp) was: 5’ -GTAAGCAGTAGTAGGTGCAGGAG-3’ and the reverse primer was: 5’-GCTTGTGTGTGTTCCAC CA-30. For GAPDH (146 bp), the forward primer was: 5’ -TCATGGTGGTGAACCAGAA-3’ and the reverse primer was 5’ -GCATGACTGTGCATGGATGAG-3.’ We normalized the expression of each targeted gene to GAPDH (ΔCt), which was our housekeeping gene. Values for gene expression were then calculated using the ^ΔΔ^Ct method with the formula: R.Q. = 2^−ΔΔCt^. TaKaRa (Kyoto, Japan) provided the SYBR^®^ Premix Ex Taq™ used for PCR amplification under the given parameters: 30 seconds at 95°C, then at 95°C again for forty cycles of 5 seconds each, followed by 30 seconds at 60°C. After that, a 15-second separation interval at 95°C, 60 seconds at 60°C, and finally 15 seconds at 95°C. The outcomes were calculated using the averages of three different reactions.

### Sodium dodecyl sulphate polyacrylamide gel electrophoresis and western blotting

A proteinase inhibitor cocktail (Sigma, USA) was added to RIPA-lysis buffer (PBS, sodium deoxycholate (0.5%), NP40 (1%), Sodium dodecyl sulphate (SDS, 0.1%) and phenylmethylsulfonylfluoride (100μg/mL)) which was applied to isolate whole tissue or cell protein from surgical samples or cell lines for 30 minutes on ice. The supernatant was harvested from centrifuged lysates. SDS-PAGE was carried out, and the segregated proteins were moved to a fluoride polyvinylidene fluoride (PVDF) membrane. Membranes were blocked for 1 hour at room temperature with 5% powdered skimmed milk, then incubated with primary antibodies overnight at 4°C. The following primary rabbit antibodies were diluted at 1:1000 (Cell Signaling Technology, Massachusetts, USA): anti-Lamin B1, anti-β-catenin and anti-FOXM1. Rabbit anti-GAPDH, also from Cell Signaling, was diluted at 1:3000. Mouse anti-DEPDC1 (1:200) was from Santa Cruz Biotech, Texas, USA). Abcam supplied anti-mouse secondary IgG H&L antibodies labelled with horseradish peroxidase (HRP, diluted 1:2000) incubated with the membrane for one hour. Bio-Rad (California, USA) supplied the Clarity Western ECL Blotting Substrates used to visualize protein locations on the membranes developed with the ChemiDoc XRS+ Bio-Rad imaging system. Our loading controls were GAPDH and Lamin B1.

### Measurement of cell colony forming capacity

Cells were seeded in 6-well plates 24 hours after transfection to examine colony-forming capacity, then incubated for approximately 14 days. Crystal violet (0.1%) and paraformaldehyde (4%) were employed to stain and fix the cells for 15 minutes at room temperature. The cell colonies were photographed and counted.

### Cell counting kit-8

We used Cell counting kit-8 (CCK-8) assays acquired from Dojindo Laboratories (Kumamoto, Japan) to measure cell proliferation. For CCK-8 assays, a 96-well plate was filled with equivalent numbers of the stated OSCC cells. The CCK-8 was used to test absorbance every 22 hours per the manufacturer’s instructions.

### Wound healing assessment

The end of a pipette was used to injure cells growing in monolayers. PBS was used to rinse off non-adhering cells before adding fresh media. Cell migration was measured as the decline in the area of the wounded region in each field image. We photographed a minimum of three fields for each measurement on each occasion, and ImageJ software was used to measure the area.

### Transwell migration assay

A Transwell assay was utilized to quantify cell migration capability. A 24-well plate separating the top and bottom chambers were used to house the Transwell chamber fitted with a polycarbonic membrane (8 μm pores, Costar, Corning, USA). The top chamber was loaded with 1 × 10^5^ OSCC cells which migrated towards fetal bovine serum (20%) in RPMI-1640 medium added to the lower chamber. Following 24 hours of incubation at 37°C, crystal violet (0.1%) was added for half an hour to stain cells in the lower chamber. Migrant cell pictures were taken under a microscope.

### Tumour growth in nude mice *in vivo*

The Beijing Critical River Laboratory provided the nude BALB/C mice (weigh t= 18.0 ± 2 g, age = 6 weeks) and were randomly divided into the stated groups. Grouped mice were injected subcutaneously with cells expressing controls or shRNA. A Vernier calliper was used to record the tumour volume (TVol) every couple of days using the equation TVol (mm^3^) = (x × y^2^)/2, where y and x represent the smallest and largest diameters. Tumours were scheduled for further testing after weighing and removal, which occurred when all animals were slaughtered 24 days after injection. At necropsy, animals were sacrificed by CO_2_ exposure followed by cervical dislocation. All procedures involving these animals were conducted in compliance with the Institutional Animal Care and Use Committee of Qingdao Municipal Hospital. The facilities and laboratory animal program of Qingdao Municipal Hospital are accredited by the Association for the Assessment and Accreditation of Laboratory Animal Care.

### Co-immunoprecipitation assay

In 12-well plates, HEK293 T cells were cultured at 1.5 × 10^5^ cells per well and incubated for 24 hours in RPMI-1640, as outlined above. Lipofectamine 3000 was applied to transfect the cells for 48 hours with DEPDC1-V5 and FOXM1-Flag plasmids. Cells were subjected to 500 μL of RIPA lysis buffer and then a protease inhibitor mixture (Thermo Science, Ontario, Canada) at 4°C for 30 minutes. For 10 minutes, the lysates were centrifuged at 4°C at 10,000 g, and 450 μL of the supernatant was reserved for the following step, whereas 50 μL was added to the 2 × SDS loading buffer as a complete extract to measure levels of the desired protein. Anti-DEPDC1 (Santa Cruz Biotech, Texas, USA), FOXM1, normal IgG, anti-V5 and anti-FLAG (all monoclonal antibodies were acquired from Cell Signaling Technology, Massachusetts, USA) were incubated with the 450 μL of supernatant while rotating at 4° C for 1 hour rotating before overnight incubation with protein G beads (Pierce, Illinois, USA) with further rotation at 4°C. Brief centrifugation at 4°C at 10,000 g then pelleted the beads, treated with 4 × RIPA buffer washes and used for Western blotting in 2 × SDS lysis buffer (50 μL).

### Co-habitation examination using immunofluorescence

The overnight cell growth on coverslips at 4°C was followed by a 1h blocking incubation with 5% milk, then incubation with the following antibodies, all diluted at 1:100: DEPDC1, Flag and FOXM1. Anti-V5 primary antibody from Cell Signaling was used at 1:50 dilution. 4’,6-diamidino-2-phenylindol (DAPI) and either Alexa Fluor 555 goat anti-mouse Ig G or Alexa Fluor 488 goat anti-mouse IgG (both diluted 1:1000, Cell Signaling Technology, Massachusetts, USA)) were subsequently applied to the coverslips. The TCS SP5 confocal microscope from Leica Microsystems (Germany) was used to obtain cell photographs.

### Statistical analysis and data presentation

The visual presentation was done with GraphPad Prism 6.0 software, while the statistical tests were performed with Statistical Package for Social Sciences (SPSS v.18.0) software. The relationship between the patient’s clinical pathology and the expression of DEPDC1 was studied by the Chi-square test. Using a Spearman rank correlation coefficient, we examined bivariate relationships between study variables. The Kaplan-Meier method was used to construct survival curves, and the logarithmic rank test was applied to compare them. The results are presented as means ± standard deviation, and the data were analyzed using Tukey’s *post hoc* test or the student’s *t-test.* Statistically significant differences were considered at probability (P) values of * P < 0.05.

## Results

### DEPDC1 overexpression is associated with poor OSCC prognosis

**Figure 1:**
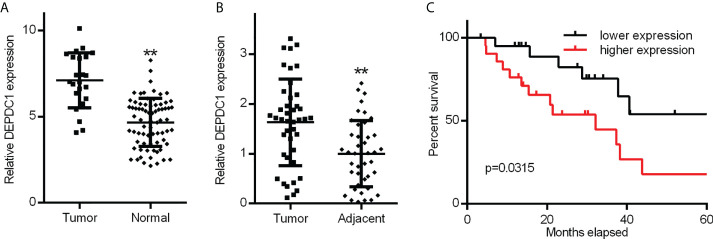
Increased OSCC tissue expression of DEPDC1. **(A)** Discovery in OSCC tissue samples of DEPDC1 expression *via* qRT-PCR by GSE31056 dataset. (normal n = 73, tumor n = 23). **(B)** DEPDC1 mRNA expression identification in OSCC tissue and neighbouring qRT-PCR samples (n = 42). **(C)** The general survival (O.S.) rate by Kaplan-Meier analysis is shown for OSCC patients with low or high DEPDC1 expression. (** P < 0.01). The data are presented as the mean ± SD. Replicate assays were performed three times.

To determine whether DEPDC1 was involved in OSCC development and progression, DEPDC1 modifications were initially examined in the GSE31056 microarray data set collected from GEO comparing OSCC and non-tumour tissues (25). The results revealed that DEPDC1 was downregulated in the GEO datasets (**Figure 1A**).

Three online sources, including GEPIA2, UALCAN and OncoLnc, were consecutively used to analyze the level of mRNA expression of DEPDC1 in human patients. Analysis of DEPDC1 expressions in human tumour tissues from the UALCAN database indicated that SSRP1 is upregulated in several tumour tissues (**Supplementary Figure 1A**). In addition, in normal tissue, HNSC and TCGA RNA sequencing data showed through GEPIA2 and UALCAN analysis that expression of DEPDC1 is upregulated in tumour tissues compared to normal tissue (**Supplementary Figures 1B, D**), whereas stages were significantly associated with DEPC1 expression (**Supplementary Figure 1C**). Moreover, Kaplan-Meier survival analysis revealed that patients with elevated levels of DEPDC1 had a poor overall (O.S.) survival relative to those with reduced levels (**Supplementary Figure 1E**).

To confirm the potential therapeutic utility of DEPDC1 expression, 42 samples were obtained for this study, including normal neighbouring and OSCC tissues. The levels of DEPDC1 expression in non-cancerous and OSCC tissues were compared. The levels were greater in OSCC tissues than in neighbouring non-cancerous ones, as revealed by qRT-PCR assay results (**Figure 1B**). The detailed clinical characteristics, including diagnosis at age, gender, lymphatic metastasis, blood glucose and TNM Stage, are shown in **Table 1**. All patients were between 35 and 82 years of age (57.47 ± 11.03 years). **Table 2** summarizes the associations between DEPDC1 expression and clinicopathological features. There was a significant correlation between the DEPDC1 expression and the lymphatic metastasis parameters (P = 0.030), TNM stage (P = 0.032), while there was no correlation between the DEPDC1 and other parameters including age (P = 0.204), gender (P = 0.153), blood glucose (P = 0.432), lymph node metastasis (P = 0.773), TNM stage (P = 0.861), radiotherapy (P = 0.624), chemotherapy (P = 0.569), relapse (P = 0.174), differentiation (P = 0.514).

**Table 1:** Clinicopathological characteristics of OSCC patient samples.

Characteristics	No. of case (%)
**Age**	
<60	16 (38.1)
≥60	26 (61.9)
**Gender**	
Male	37 (88.1)
Female	5 (11.9)
**Lymphatic metastasis**	
Yes	32 (76.2)
No	10 (23.8)
**Blood glucose (mmol/L)**	
Normal	34 (81.0)
High	8 (19.0)
**TNM stage**	
stage I	28 (66.7)
stage II	4 (9.5)
stage III	10 (23.8)

OSCC, oral squamous cell carcinoma; TNM, tumor-node-metastasis.

**Table 2:** Correlation between DEPDC1 expression and clinicopathologic characteristics of OSCC patients.

Characteristics DEPDC1	DEPDC1 expression		
	Low no. cases	High no. cases	p value
**Age**			
<60	10	6	0.204
≥60	11	15	
**Gender**			
Male	17	20	0.153
Female	4	1	
**Lymphatic metastasis**			
Yes	2	8	**0.030**
No	19	13	
**Blood glucose (mmol/L)**			
Normal	18	16	0.432
High	3	5	
**TNM stage**			
stage I	18	10	**0.032**
stage II	1	3	
stage III	2	8	

P values were calculated using chi-square test. Bold numbers indicate significant differences (P < 0.05). OSCC oral squamous cell carcinoma, TNM tumor-node-metastasis.

Spearman’s analysis of the correlation between DEPDC1 and clinicopathological features revealed that the expression of DEPDC1 was significantly associated with lymphatic metastasis (P = 0.030) and TNM stages (P = 0.008). Additionally, we examined the relative risks indicated by DEPDC1 in the OSCC prognostic. The Cox regression analysis was performed to determine if DEPDC1 could be a potential risk factor. As shown in **Table 3**, high expression of DEPDC1 and blood glucose was associated with a significantly elevated risk of death in OSCC patients (P = 0.039 and P = 0.022) compared to those with low DEPDC1 expression by univariate Cox regression analysis (**Table 4**). Multivariate Cox regression analysis revealed that blood glucose could be a factor in predicting poor survival (P = 0.019) (**Table 4**). These results indicate a significant correlation between the expression of DEPDC1 with the prognosis of OSCC.

**Table 3:** Spearman analysis of correlation between DEPDC1 and clinicopathological.

Variables	DEPDC1 expression level	
	Spearman correlation	p value
**Age (years, <60 vs.** ≥60)	0.196	0.213
**Gender (male/female)**	-0.221	0.16
**Lymphatic metastasis (yes/no)**	0.335	**0.03**
**Blood glucose (normal vs high)**	0.121	0.444
**TNM stage (I+II vs III)**	0.402	**0.008**

Bold numbers indicate significant differences (P < 0.05). OSCC oral squamous cell carcinoma, TNM tumor-node-metastasis.

**Table 4:** Univariate and multivariate analyses of various prognostic parameters in patients with OSCC Cox-regression analysis.

	Univariate analysis				Multivariate analysis		
	p value	Hazard Ratio	95% confidence interval		p value	Hazard Ratio	95% confidence interval
**DEPDC1**	**0.039**	2.779	1.052-7.342		0.095	2.436	0.857-6.919
**Lymphatic metastasis (yes/no)**	0.392	1.529	0.578-4.046		0.431	1.601	0.496-5.168
**Blood glucose (normal vs high)**	**0.022**	3.316	1.188-9.257		**0.019**	4.087	1.262-13.238

Bold numbers indicate significant differences (P < 0.05). OSCC oral squamous cell carcinoma, TNM tumor-node-metastasis.

The Kaplan-Meier analysis showed that patients with reduced expression of DEPDC1 had a markedly longer overall survival (O.S.) rate (O.S.: 71.43% *vs* 38.09%) than patients with high expression of DEPDC1; taking the DEPDC1 mid-level value as the cut-off point in 42 patients (**Figure 1C**). The findings suggest that DEPDC1 may be overexpressed and associated with poor OSCC prognosis.

### DEPDC1 promotes *in vitro* OSCC cell metastasis and proliferation

**Figure 2:**
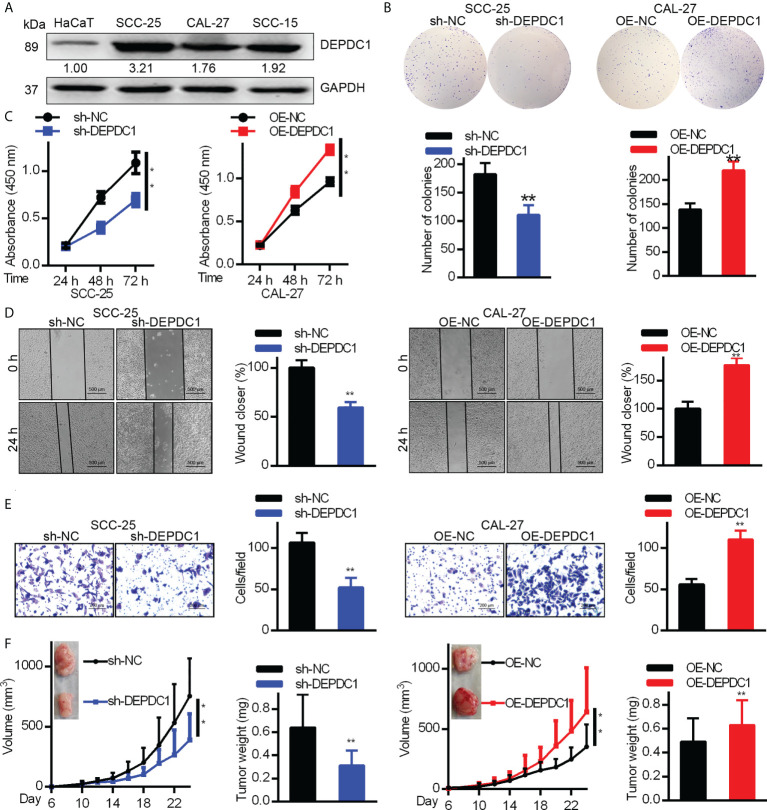
DEPDC1 promotes human OSCC cell tumorigenicity. **(A)** The expression of DEPDC1 in the HaCaT immortal keratinocyte cell line and three cell lines of OSCC (SCC-15, CAL-27 and CAL-25) was assessed with Western blot. Results were standardized to the GAPDH loading control. **(B)** SCC-25 cells had DEPDC1 knocked down, while in CAL-27 cells, it was overexpressed. **(B, C)** The colony formation study was studied by the Cell counting kit 8 (CCK-8). **(D)** A wound-healing test was performed. **(E)** Migration was measured using a Transwell assay. The figure-panels quantify and compare the number of cells going through the membrane and the proportion of wound closure. Scale bars are 500 µm **(D)** and 200 µm **(E)**. The results are shown as mean ± S.D. P < 0.05, ** P < 0.01, N = 3. **(F)** Each of 6 nude mice aged 6 weeks had 1 × 10^6^ control or DEPDC1-knockdown SCC-25 cells injected under their skin. Weights and volumes of tumours were analyzed. Similarly, 1.5 × 10^6^ control or DEPDC1-knockdown CAL-27 cells were injected under the skin of 6 nude mice. The weights and volume of the tumours were analyzed. For **(F)** A student’s *t-test* was used to compare tumor weights between 2 groups, whereas the two-way ANOVA was performed for larger groups. The data are presented as the mean ± SD, and are representative of at least 3 independent experiments.

In order to determine DEPDC1’s biological function in OSCC cells, we analyzed DEPDC expression levels in the HaCaT immortalized keratinocyte cell line and three OSCC cell lines (**Figure 2A**). According to our findings, in each OSCC cell line, DEPDC1 expression was greater than that of HaCaT cells. CAL-27 had the lowest concentration of DEPDC1, whereas SCC-25 had the highest concentration. Then, DEPDC1 was downregulated in SCC-25 cells and increased in CAL-27 cells, respectively (**Supplementary Figures 2A, B**). To investigate the biological function of DEPDC1 on OSCC progression, CCK-8 and colony formation assays were performed. Compared to the controls transfected with sh-NC, the SCC-25 cells transfected with sh-DEPDC1 had considerably lower cell viability (**Figure 2B, C**) (negative control). The propagation of OV-DEPDC1-transfected CAL-27 cells was better than that of OV-NC-transfected CAL-27 cells (**Figure 2B, C**).

We conducted Transwell and wound healing experiments to investigate how OSCC cell migration was affected by DEPDC1. Wound healing test results showed that DEPDC1 ectopically expressing CAL-27 cells had faster closure of wounds than control cells (**Figure 2D**), whereas the abolition of SCC-25 cell DEPDC1 expression delayed closure of wounds relative to controls (**Figure 2D**). The knockdown of DEPDC prevents the migration of SCC-25 cells, as demonstrated by the Transwell test, which also showed that DEPDC1 overexpression caused cell migration (**Figure 2E**). We next investigated the impact of DEPDC1 on nude mouse tumour growth to obtain confirmation that DEPDC1 enhanced tumour progression and found that DEPDC1 knockdown shrank the tumours (**Figure 2F** **, left**). In contrast, increased *in vivo* tumour size was observed following the overexpression of DEPDC1 in CAL-27 tumours (**Figure 4F**, **right**). Together, these results showed that in OSCC, DEPDC1 plays a cancer-inducing role *in vivo* and *in vitro*.

### DEPDC1 associates with FOXM1

**Figure 3:**
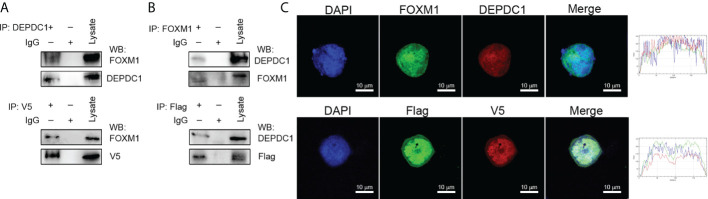
DEPDC1 associates with FOXM1. **(A)** FOXM1-expressing plasmids tagged with FLAG and DEPDC1 plasmids with V5 tags were both transfected into HEK293 T cells. Co-immunoprecipitation of transfected cell lysates using anti-V5 or anti-DEPDC1 antibodies bound to beads. Western blotting was then used to identify DEPDC1 is labelled with V5 using anti-V5 or anti-DEPDC1 antibodies. **(B)** Reverse immunoprecipitation. We transfected HEK293T cells with controls or FLAG-bound FOXM1 and V5-bound DEPDC1. Anti-V5 or anti-FOXM1 antibodies were used to Co-immunoprecipitate cell lysates. Anti-FLAG or anti-FOXM1 antibodies were used for Western blotting to detect immunoprecipitated products **(C)**. The subcellular sites of DEPDC1-V5 and FOXM1-Flag were transfected into SCC-25 cells. One day after transfection, cells were fixed, penetrated and incubated with green FLAG, red V5, green FOXM1 and red DEPDC1 Confocal microscopy were performed to analyze nuclei stained with DAPI (blue). Graphs of the intensity profile are shown at the bottom of the image (x-axis, length [in microns]; y-axis, intensity). The bar is set to scale: 10 µm. The data are representative of at least 3 independent experiments.

The GEPIA2 website was utilized to investigate related genes, find those associated with DEPDC1 and show the biomolecular mechanisms by which DEPDC1 improved OSCC tumorigenicity. **Figure 3A** shows the FOXM1-associated proteins that have been discovered. Because of FOXM1’s crucial role in carcinogenesis in our earlier study, we decided to investigate it further (20). Following Genecards (https://www.genecards.org/) to analyze protein positions, the two potentially associated protein location sites were strongly coincidental (**Supplementary Figure 3B**). Therefore, a co-immunoprecipitation approach was used with cultured cell lysates to confirm the presumed physical interactions between FOXM1 and DEPDC1. The vectors DEPDC1-V5 and FOXM1-Flag were generated (**Supplementary Figure 3C**). SCC-25 cells revealed the interaction between endogenous FOXM1 and DEPDC1 (**Figure 3B**). Moreover, DEPDC1 (red) and FOXM1 (green) immunofluorescence staining showed that the two proteins co-habited in the nucleus (**Figure 3C**). These findings suggest that DEPDC1 is a validated interacting protein for FOXM1.

### DEPDC1 accelerates OSCC cell growth *via* regulating FOXM1

**Figure 4:**
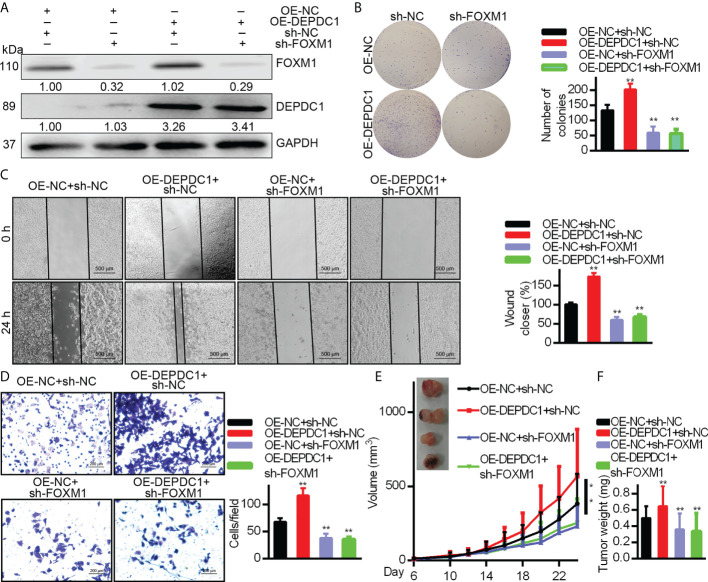
DEPDC1 facilitates the OSCC cell migration and growth via FOXM1. DEPDC1-over-expressing CAL-27 cells had FOXM1 knocked down. The indicated antibodies were used for Western blotting cell lysates **(A)**. Colony formation **(B)**, wound healing (500 μm scale bar) **(C)**, and Transwell (100 μm scale bar) assays **(D)** have been studied in cell growth and migration. The results shown for (**B–D**) are mean ± S.D. N = 3 separate tests. Data were analyzed with the student’s *t-test*. In the CAL-27 cells, FOXM1 was knocked down with high or low over-expression of DEPDC1 and 1.5 × 10^6^ cells were injected under the skin of 6-week nude mice (n = 6). The development of tumours was studied. The data are shown as mean ± S.D. The student’s *t-test* **(F)**, Two-way ANOVA procedure **(E)** and been used to assess statistical significance. The data are representative of at least 3 independent experiments * P < 0.05, ** P < 0.01..

As mentioned above, DEPDC1 was verified to play critical roles in promoting growth in OSCC cells. We suppressed FOXM1 endogenous expression in CAL–27 cells overexpressing DEPDC1 to see if DEPDC1-mediated FOXM1 regulation promotes tumour growth. We determined that DEPDC1 inhibited cell migration and proliferation under these conditions (**Figures 4A–D**). To further explore whether DEPDC1 promoted OSCC growth, the xenograft experiments were established in nude mice. Xenograft experiments frequently indicated that the consequences of over-expressing DEPDC1 were rescued by suppressing FOXM1 levels, resulting in the reduced progression of tumours (**Figures 4E, F**). Together, these results show that DEPDC1 stimulated tumour development by FOXM1 regulation.

### DEPDC1 activates Wnt signaling in OSCC

**Figure 5:**
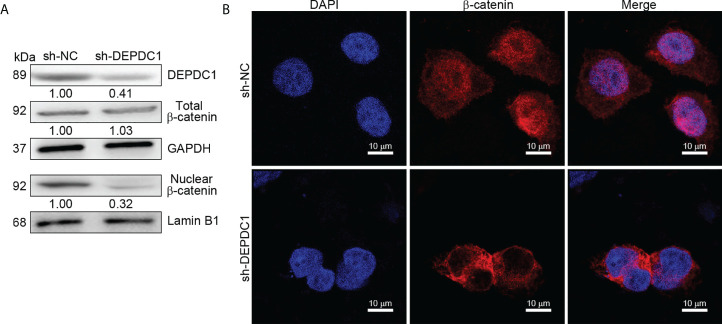
DEPDC1 downregulations in oral squamous cell carcinoma inhibit the Wnt/β-catenin signaling pathway. **(A)** DEPDC1 has been tested for its effect on the Wnt/β-catenin signaling pathway in SstainingstainingstainingCC-25 cells using a Western blot assessment. **(B)** The nuclear translocation of β-catenin was mediated by DEPDC1-immunofluorescence-stained β-catenin in sh-DEPDC1 treatment of SCC-25 cells. The data are presented as the mean ± SD, and are representative of at least 3 independent experiments.

Since our previous study revealed that a positive correlation exists between FOXM1 and Wnt signaling, we decided to focus efforts on FOXM1 since it is a critical transcriptional coactivator of Wnt signaling, which is tightly linked to tumor growth and metastasis (17). DEPDC1 has already been reported to regulate the Wnt/β-catenin pathway (10). Additionally, Wnt/β-catenin stimulation can increase OSCC cell invasion and proliferation (26). As a result, we hypothesized that DEPDC1 might promote OSCC malignancy by initiating a Wnt/β-catenin signaling cascade. Western blot experiments were used to test this theory. Results showed that SCC-25 cell DEPDC1-downregulation decreased protein expression in the Wnt/β-catenin pathway, particularly the aggregation of β-Catenin in the nucleus (**Figure 5A**). Furthermore, immunofluorescence staining data shows that after DEPDC1 has been downregulated, β-catenin moves to the cytoplasm from the nucleus in SCC-25 cells (**Figure 5B**). When taken together, the results demonstrate that DEPDC1 employs Wnt/β-catenin signaling to promote malignant OSCC progression.

## Discussion

Our findings provide compelling evidence for DEPDC1’s important function in OSCC development regulation. DEPDC1 promotes the development and proliferation of human OSCC cells *via* Wnt/β-catenin signaling. DEPDC1 interacted with FOXM1, which led to the nuclear localization of β-catenin, the most important indicator for Wnt pathway activation. The level of DEPDC1 expression was also highly correlated with the level of FOXM1 expression in human OSCCs in online databases. DEPDC1 has since been discovered to play a role in cancer progression and is widely regarded as a possible oncogene.

The most common cancer type in which DEPDC1 was shown to play a function was bladder cancer (8, 27). Studies have shown that DEPDC1 is significantly elevated in bladder cancer and is important for cancer cell proliferation (8). DEPDC1 was revealed as one of the most activated genes in breast cancer by analyzing microarray data (28). The TCGA, open-access data, are used to evaluate the function of breast cancer genes, providing scientists with a valuable research tool and novel techniques for cancer prevention, treatment, and diagnostics (29). This report examined DEPDC1’s transcription profiles in the GEO and TCGA datasets. Our findings showed that levels of expression of DEPDC1 protein and mRNA in OSCC tissue were considerably increased, and a progressive improvement in the tumour stage was recorded. DEPDC1 expression was lower in normal adjacent tissue relative to OSCC tissue. In addition, several studies have found that longer survival in patients with multiple myeloma and hepatocellular carcinoma was significantly correlated with a lower DEPDC1 expression, suggesting that DEPDC1 may be a new diagnostic marker (30, 31). According to these reports, increased DEPDC1 in OSCC cancer significantly correlated with shorter patient survival times.

Cancer development and proliferation are promoted by abnormal gene expression (32). In nasopharyngeal carcinomas, suppressing DEPDC1 slows the cell cycle and significantly reduces invasion, migration, and proliferation (13). A separate study showed that DEPDC1 inhibits A549 cell proliferation by blocking cell apoptosis (33). Overexpression of DEPDC1 constantly increased the possibility of cancer development and proliferation in breast cancer cells, which was prevented by its deletion. In addition, TCGA datasets revealed that DEPDC1 was a new, highly expressed gene contributing to HNSC migration and proliferation. Together, the results suggest that DEPDC1 mediates a cancer-inducing function in OSCC cells in addition to facilitating OSCC progression through modulating the OSS cell growth environment. In OSCC cancer cells, correlations between DEPDC1 and FOXM1 were also discovered. FOXM1 is a critical regulator of chemoresistance, metastasis, and oncogenesis (16). Our previous reports found that FOXM1 is overexpressed in OSCC cells (17).

Nonetheless, the expression regulators upstream of FOXM1 remain unknown. Merlin has been demonstrated to regulate FOXM1 expression in pancreatic tumours by ubiquitin-labelling and degradation of FOXM1 (34). Here, we also observed that DEPDC1 interacts with FOXM1. It was recently reported that DEPDC1 is mainly found in cancer cell nuclei (8). However, the accumulation of FOXM1 in the nucleus attracts β-catenin to the Wnt gene promoter, thereby inducing Wnt signaling (35). By managing the structural changes in FOXM1, DEPDC1 can make it more stable. Despite these facts, a better understanding of the complicated mechanisms involved in DEPDC1-mediated FOXM1 stability regulation is needed.

The molecular processes behind the function of DEPDC1 remain uncertain. In cancer, Wnt/β-catenin pathways are usually disrupted and are key OSCC tumorigenesis mediators (36). Nuclear migration and stability of β-catenin are important processes for the continuous stimulation of this signaling. The results from gene enrichment examination suggest that general and WNT/β-catenin-specific cancer signaling pathways related to HCC cancer genes had a positive correlation with DEPDC1 expression (10). In earlier studies, Zhang et al. showed that nuclear translocation of β-catenin was enhanced by direct FOXM1 binding (37). This is the first time that a blocked Wnt/β-catenin signal and β-catenin nuclear translocation have been reported in OSCC cells due to DEPDC1 downregulation. Since Wnt/β-catenin signaling is an important contributor to OSCC development and spread, identifying pathological molecular mechanisms behind OSCC will be aided by discovering a DEPDC1/FOXM1/β-catenin signaling complex.

## Conclusion

These findings show that DEPDC1 overexpression is linked to poor clinical outcomes and tumour progression in OSCC patients. FOXM1 interacts with DEPDC1, which causes cancer in OSCC cells, according to these fundamental *in vitro* data. Furthermore, this study describes a novel regulatory signaling pathway driven by DEPDC1 that enhances cancer characteristics by leveraging the Wnt/β-catenin OSCC. Our findings add to our understanding of the pathogenesis of OSCC cancer tumorigenesis.

## Data availability statement

The original contributions presented in the study are included in the article/**Supplementary Material**. Further inquiries can be directed to the corresponding authors.

## Ethics statement

This study was approved by the Ethics and Research Committee of Qingdao Municipal Hospital and conducted according to the ethical guidelines of the 1975 Declaration of Helsinki. The animal study was reviewed and approved by the Institutional Animal Care and Use Committee of Qingdao Municipal Hospital. Approval reference number QMHAS20180812. Written informed consent was obtained from the individual(s) for the publication of any potentially identifiable images or data included in this article.

## Author contributions

JQ, YT, HC and RY performed most of the experiments included in this study. HC and RY wrote the first draft of the manuscript. LL completed and formatted the manuscript for submission. JY and ZC helped in analyzing the data. All authors contributed to the article and approved the submitted version.

## Funding

This work was supported by Natural Science Foundation of Zhejiang Province of China (LGF19H160014) and Natural Science Basic Research Program Shaanxi Province (2020JM-008).

## Acknowledgments

We appreciate researchers who have developed and maintained public databases such as GEPIA2, UALCAN, and OncoLnc (http://www.oncolnc.org/), which will accelerate the understanding and treatment of human cancer.

## Conflict of interest

The authors declare that the research was conducted in the absence of any commercial or financial relationships that could be construed as a potential conflict of interest.

## Publisher’s note

All claims expressed in this article are solely those of the authors and do not necessarily represent those of their affiliated organizations, or those of the publisher, the editors and the reviewers. Any product that may be evaluated in this article, or claim that may be made by its manufacturer, is not guaranteed or endorsed by the publisher.

## Supplementary material

Supplementary Figure 1 Expression and overall survival (O.S.) of DEPDC1 family in HNSC patients from the GEPIA, UALCAN and OncoLnc databases. **(A)** UALCAN database displays DEPDC1 is in a high expression status in multiple tumour types. **(B)** Expression of DEPDC1 in patients with HNSC in the UALCAN database. **(C)** DEPDC1 is associated with clinical stages (UALCAN) in HNSC. **(D)** Expression of DEPDC1 in patients with HNSC in the GEPIA database. **(E)** HNSC Patients in the OncoLnc sample were assessed using The Kaplan-Meier approach, which was used to classify patients into a group of high expression with ≥25% above the median. Meanwhile, the low expression had ≤25% less than the median.

Supplementary Figure 2 SCC-25 cell DEPDC1 was knocked down and overexpressed in the CAL-27 cells. **(A)** Detection of DEPDC1 expression in SCC-25 cells after down-regulated by sh-DEPDC1. **(B)** Detection of DEPDC1 expression in CAL-27 cells after upregulated by OE-DEPDC1. **P < 0.01. The data are presented as the mean ± SD, and are representative of at least 3 independent experiments.

Supplementary Figure 3 Identification of the DEPDC1 interacting proteins. **(A)** The FOXM1 protein expression correlated with DEPDC1 by Pearson correlation coefficient (PCC) analysis. **(B)** The intracellular location of DEPDC1 and FOXM1 was shown on the GeneCards website. **(C)** Construction map of FOXM1-Flag and DEPDC1-V5 vectors.

## References

[1] Siegel RL Miller KD Fuchs HE Jemal A. Cancer statistics, 2022. CA Cancer J Clin (2022) 72:7–33. doi: 10.3322/caac.21708 35020204

[2] Leemans CR Snijders P Brakenhoff RH. The molecular landscape of head and neck cancer. Nat Rev Cancer. (2018) 18:269–82. doi: 10.1038/nrc.2018.11 29497144

[3] Zeng H Chen W Zheng R Zhang S Ji JS Zou X. Changing cancer survival in china during 2003-15: a pooled analysis of 17 population-based cancer registries. Lancet Glob Health (2018) 6:e555–67. doi: 10.1016/S2214-109X(18)30127-X 29653628

[4] Sung H Ferlay J Siegel RL Laversanne M Soerjomataram I Jemal A. Global cancer statistics 2020: globocan estimates of incidence and mortality worldwide for 36 cancers in 185 countries. CA Cancer J Clin (2021) 71:209–49. doi: 10.3322/caac.21660 33538338

[5] Su CW Lin CW Yang WE Yang SF. Timp-3 as a therapeutic target for cancer. Ther Adv Med Oncol (2019) 11:432488009. doi: 10.1177/1758835919864247 PMC6637839 31360238

[6] Sendoel A Maida S Zheng X Teo Y Stergiou L Rossi CA. Depdc1/let-99 participates in an evolutionarily conserved pathway for anti-tubulin drug-induced apoptosis. Nat Cell Biol (2014) 16:812–20. doi: 10.1038/ncb3010 25064737

[7] Mi Y Zhang C Bu Y Zhang Y He L Li H. Depdc1 is a novel cell cycle related gene that regulates mitotic progression. Bmb Rep (2015) 48:413–8. doi: 10.5483/bmbrep.2015.48.7.036 PMC4577292 25902835

[8] Harada Y Kanehira M Fujisawa Y Takata R Shuin T Miki T. Cell-permeable peptide depdc1-znf224 interferes with transcriptional repression and oncogenicity in bladder cancer cells. Cancer Res (2010) 70:5829–39. doi: 10.1158/0008-5472.CAN-10-0255 20587513

[9] Li Y Tian Y Zhong W Wang N Wang Y Zhang Y. Artemisia argyi essential oil inhibits hepatocellular carcinoma metastasis *via* suppression of depdc1 dependent wnt/beta-catenin signaling pathway. Front Cell Dev Biol (2021) 9:664791. doi: 10.3389/fcell.2021.664791 34268303 PMC8276134

[10] Qu D Cui F Lu D Yang Y Xu Y. Dep domain containing 1 predicts prognosis of hepatocellular carcinoma patients and regulates tumor proliferation and metastasis. Cancer Sci (2019) 110:157–65. doi: 10.1111/cas.13867 PMC6317931 30417471

[11] Zhang L Du Y Xu S Jiang Y Yuan C Zhou L. Depdc1, negatively regulated by mir-26b, facilitates cell proliferation *via* the up-regulation of foxm1 expression in tnbc. Cancer Lett (2019) 442:242–51. doi: 10.1016/j.canlet.2018.11.003 30419349

[12] Gong Z Chu H Chen J Jiang L Gong B Zhu P. Depdc1 upregulation promotes cell proliferation and predicts poor prognosis in patients with gastric cancer. Cancer biomark (2021) 30:299–307. doi: 10.3233/CBM-201760 33361586 PMC12499972

[13] Feng X Zhang C Zhu L Zhang L Li H He L. Depdc1 is required for cell cycle progression and motility in nasopharyngeal carcinoma. Oncotarget. (2017) 8:63605–19. doi: 10.18632/oncotarget.18868 PMC5609947 28969015

[14] Kopanja D Chand V O'Brien EM Mukhopadhyay NK Zappia MP Islam A. Transcriptional repression by foxm1 suppresses tumor differentiation and promotes metastasis of breast cancer. Cancer Res (2022) 82:2458–71. doi: 10.1158/0008-5472.CAN-22-0410 PMC9258028 35583996

[15] Xing S Tian Z Zheng W Yang W Du N Gu Y. Hypoxia downregulated mir-4521 suppresses gastric carcinoma progression through regulation of igf2 and foxm1. Mol Cancer. (2021) 20:9. doi: 10.1186/s12943-020-01295-2 33407516 PMC7786912

[16] Kalathil D John S Nair AS. Foxm1 and cancer: faulty cellular signaling derails homeostasis. Front Oncol (2020) 10:626836. doi: 10.3389/fonc.2020.626836 33680951 PMC7927600

[17] Qiu J Zhao J Zuo A Liu L Liu Q Pan H. Lentiviral rna interference-mediated downregulation of forkhead box m1 expression suppresses growth of oral squamous cell carcinoma. vitro. Oncol Lett (2019) 17:525–31. doi: 10.3892/ol.2018.9536 PMC6313164 30655797

[18] Borhani S Gartel AL. Foxm1: a potential therapeutic target in human solid cancers. Expert Opin Ther Targets. (2020) 24:205–17. doi: 10.1080/14728222.2020.1727888 32067537

[19] Ramos-Garcia P Gonzalez-Moles MA. Prognostic and clinicopathological significance of the aberrant expression of beta-catenin in oral squamous cell carcinoma: a systematic review and meta-analysis. Cancers (Basel). (2022) 14:479. doi: 10.3390/cancers14030479 35158747 PMC8833491

[20] Bugter JM Fenderico N Maurice MM. Mutations and mechanisms of wnt pathway tumour suppressors in cancer. Nat Rev Cancer. (2021) 21:5–21. doi: 10.1038/s41568-020-00307-z 33097916

[21] Hsu CC Liao WY Chang KY Chan TS Huang PJ Chiang CT. A multi-mode wnt- and stemness-regulatory module dictated by foxm1 and aspm isoform i in gastric cancer. Gastric Cancer. (2021) 24:624–39. doi: 10.1007/s10120-020-01154-5 33515163

[22] Tang Z Kang B Li C Chen T Zhang Z. Gepia2: an enhanced web server for large-scale expression profiling and interactive analysis. Nucleic Acids Res (2019) 47:W556–60. doi: 10.1093/nar/gkz430 PMC6602440 31114875

[23] Chandrashekar DS Bashel B Balasubramanya S Creighton CJ Ponce-Rodriguez I Chakravarthi B. Ualcan: a portal for facilitating tumor subgroup gene expression and survival analyses. Neoplasia. (2017) 19:649–58. doi: 10.1016/j.neo.2017.05.002 PMC5516091 28732212

[24] Zheng H Zhang G Zhang L Wang Q Li H Han Y. Comprehensive review of web servers and bioinformatics tools for cancer prognosis analysis. Front Oncol (2020) 10:68. doi: 10.3389/fonc.2020.00068 32117725 PMC7013087

[25] Reis PP Waldron L Perez-Ordonez B Pintilie M Galloni NN Xuan Y. A gene signature in histologically normal surgical margins is predictive of oral carcinoma recurrence. BMC Cancer. (2011) 11:437. doi: 10.1186/1471-2407-11-437 21989116 PMC3198722

[26] Qiao C Qiao T Yang S Liu L Zheng M. Snhg17/mir-384/elf1 axis promotes cell growth by transcriptional regulation of ctnnb1 to activate wnt/beta-catenin pathway in oral squamous cell carcinoma. Cancer Gene Ther (2022) 29:122–32. doi: 10.1038/s41417-021-00294-9 33531646

[27] Wang Y Wu J Luo W Zhang H Shi G Shen Y. Alpk2 acts as tumor promotor in development of bladder cancer through targeting depdc1a. Cell Death Dis (2021) 12:661. doi: 10.1038/s41419-021-03947-7 34210956 PMC8249393

[28] Colak D Nofal A Albakheet A Nirmal M Jeprel H Eldali A. Age-specific gene expression signatures for breast tumors and cross-species conserved potential cancer progression markers in young women. PLoS One (2013) 8:e63204. doi: 10.1371/journal.pone.0063204 23704896 PMC3660335

[29] Tomczak K Czerwinska P Wiznerowicz M. The cancer genome atlas (tcga): an immeasurable source of knowledge. Contemp Oncol (Pozn). (2015) 19:A68–77. doi: 10.5114/wo.2014.47136 PMC4322527 25691825

[30] Zhang L Li Y Dai Y Wang D Wang X Cao Y. Glycolysis-related gene expression profiling serves as a novel prognosis risk predictor for human hepatocellular carcinoma. Sci Rep (2021) 11:18875. doi: 10.1038/s41598-021-98381-2 34556750 PMC8460833

[31] Zhang J Liu X Zhou W Lu S Wu C Wu Z. Identification of key genes associated with the process of hepatitis b inflammation and cancer transformation by integrated bioinformatics analysis. Front Genet (2021) 12:654517. doi: 10.3389/fgene.2021.654517 34539726 PMC8440810

[32] Oren Y Tsabar M Cuoco MS Amir-Zilberstein L Cabanos HF Hutter JC. Cycling cancer persister cells arise from lineages with distinct programs. Nature. (2021) 596:576–82. doi: 10.1038/s41586-021-03796-6 PMC9209846 34381210

[33] Bolomsky A Heusschen R Schlangen K Stangelberger K Muller J Schreiner W. Maternal embryonic leucine zipper kinase is a novel target for proliferation-associated high-risk myeloma. Haematologica. (2018) 103:325–35. doi: 10.3324/haematol.2017.172973 PMC5792277 29122991

[34] Quan M Cui J Xia T Jia Z Xie D Wei D. Merlin/nf2 suppresses pancreatic tumor growth and metastasis by attenuating the foxm1-mediated wnt/beta-catenin signaling. Cancer Res (2015) 75:4778–89. doi: 10.1158/0008-5472.CAN-14-1952 PMC4651817 26483206

[35] Chen Y Li Y Xue J Gong A Yu G Zhou A. Wnt-induced deubiquitination foxm1 ensures nucleus beta-catenin transactivation. EMBO J (2016) 35:668–84. doi: 10.15252/embj.201592810 PMC4801947 26912724

[36] Xie J Huang L Lu YG Zheng DL. Roles of the wnt signaling pathway in head and neck squamous cell carcinoma. Front Mol Biosci (2020) 7:590912. doi: 10.3389/fmolb.2020.590912 33469547 PMC7814318

[37] Zhang N Wei P Gong A Chiu WT Lee HT Colman H. Foxm1 promotes beta-catenin nuclear localization and controls wnt target-gene expression and glioma tumorigenesis. Cancer Cell (2011) 20:427–42. doi: 10.1016/j.ccr.2011.08.016 PMC3199318 22014570

