# Supplementary figures and images for: FOXM1 is regulated by DEPDC1 to facilitate development and metastasis of oral squamous cell carcinoma
Source: Front Oncol. 2022 Aug 22;12:815998. doi: 10.3389/fonc.2022.815998 (PMC9443502; doi:10.3389/fonc.2022.815998)

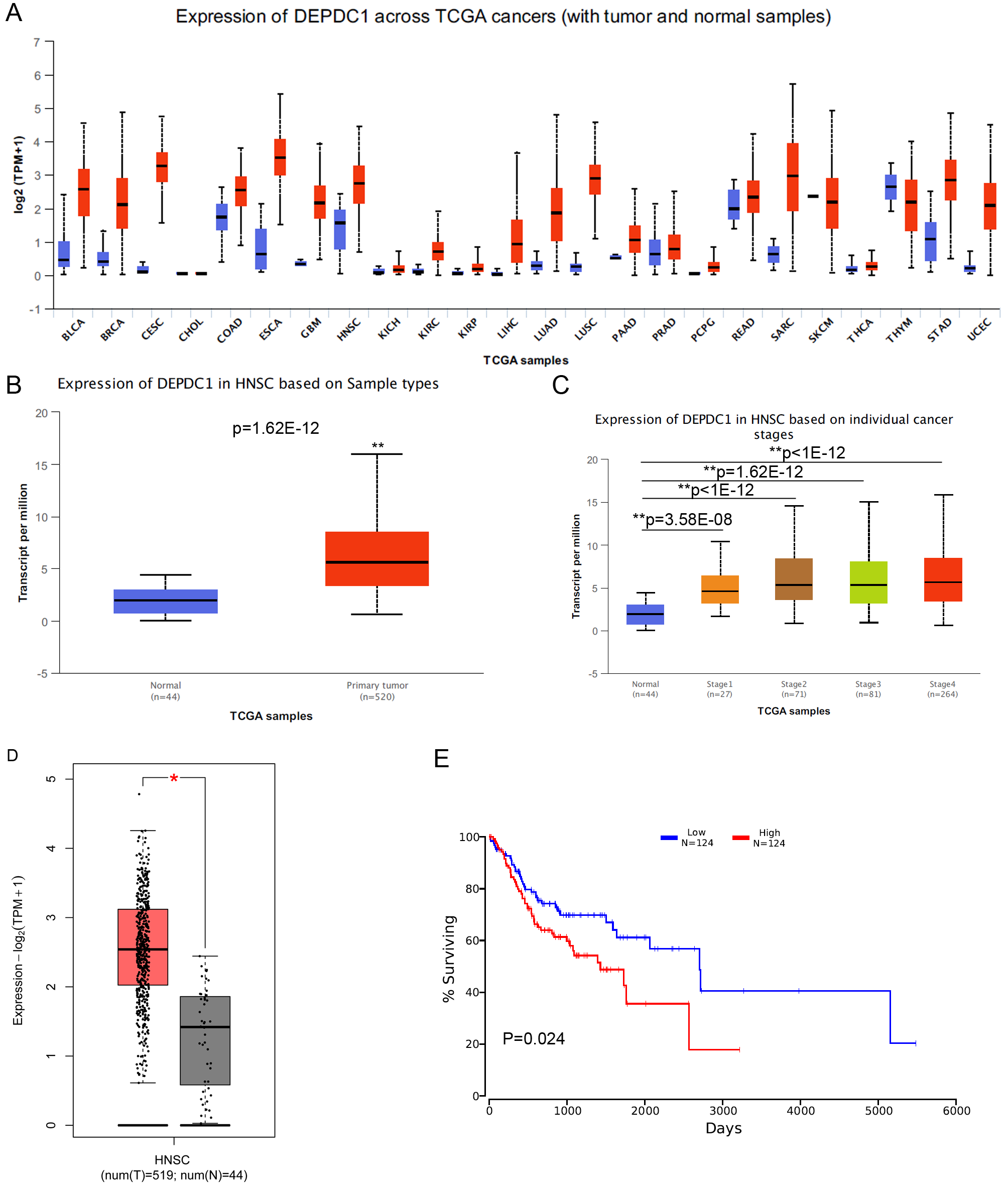

Supplement: Supplementary Figure 1 — Expression and overall survival (O.S.) of DEPDC1 family in HNSC patients from the GEPIA, UALCAN and OncoLnc databases. (A) UALCAN database displays DEPDC1 is in a high expression status in multiple tumour types. (B) Expression of DEPDC1 in patients with HNSC in the UALCAN database. (C) DEPDC1 is associated with clinical stages (UALCAN) in HNSC. (D) Expression of DEPDC1 in patients with HNSC in the GEPIA database. (E) HNSC Patients in the OncoLnc sample were assessed using The Kaplan-Meier approach, which was used to classify patients into a group of high expression with ≥25% above the median. Meanwhile, the low expression had ≤25% less than the median. [file Image_1.tif]

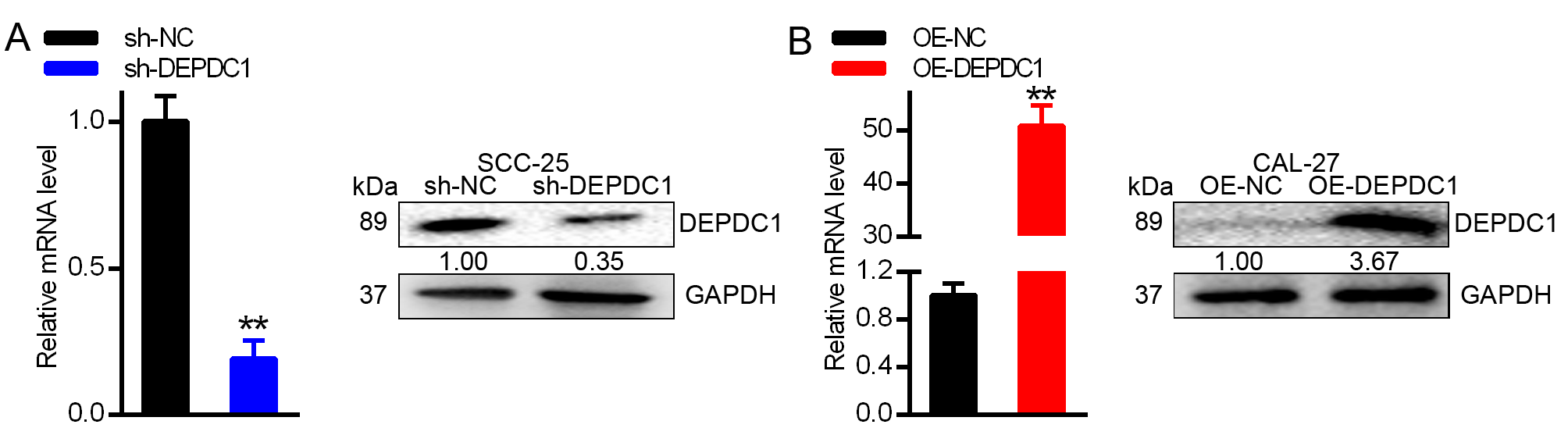

Supplement: Supplementary Figure 2 — SCC-25 cell DEPDC1 was knocked down and overexpressed in the CAL-27 cells. (A) Detection of DEPDC1 expression in SCC-25 cells after down-regulated by sh-DEPDC1. (B) Detection of DEPDC1 expression in CAL-27 cells after upregulated by OE-DEPDC1. **P < 0.01. The data are presented as the mean ± SD, and are representative of at least 3 independent experiments. [file Image_2.tif]

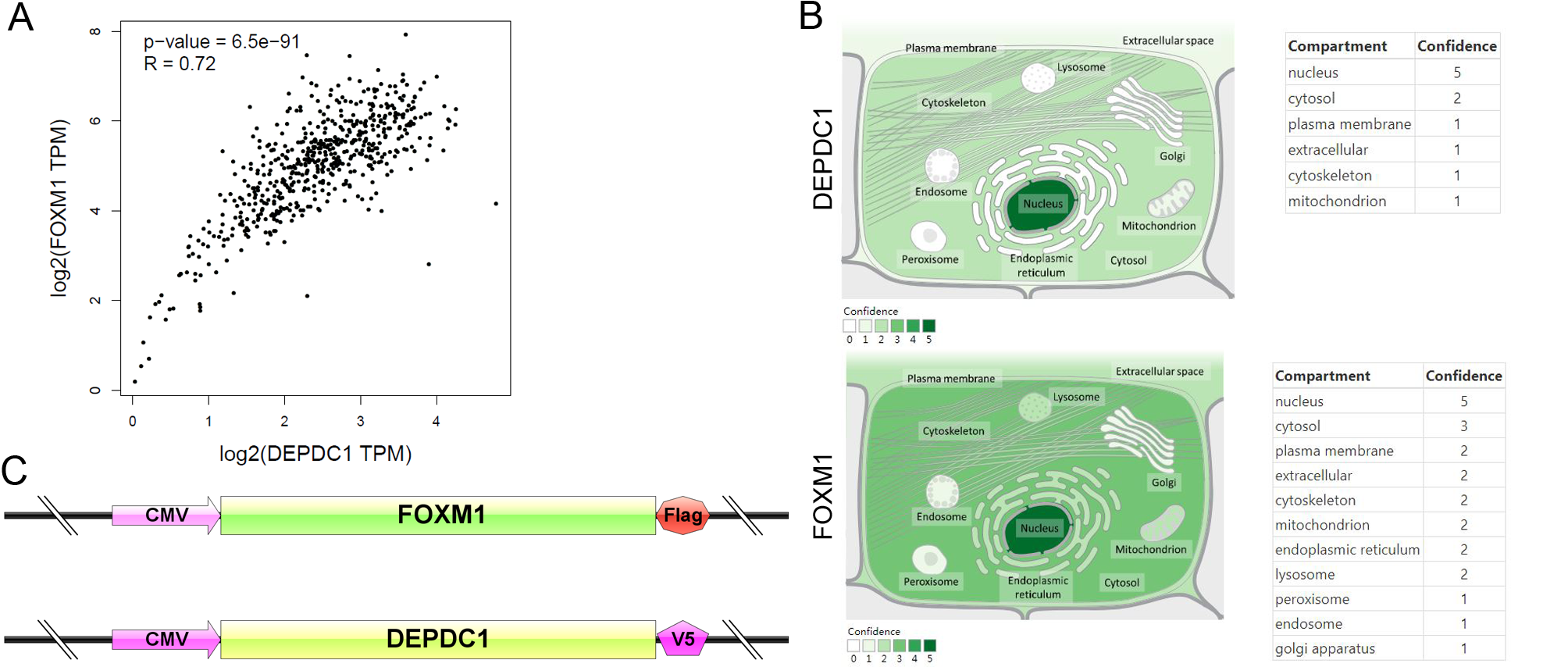

Supplement: Supplementary Figure 3 — Identification of the DEPDC1 interacting proteins. (A) The FOXM1 protein expression correlated with DEPDC1 by Pearson correlation coefficient (PCC) analysis. (B) The intracellular location of DEPDC1 and FOXM1 was shown on the GeneCards website. (C) Construction map of FOXM1-Flag and DEPDC1-V5 vectors. [file Image_3.tif]
